# The effect of chronic tianeptine administration on the brain mitochondria: direct links with an animal model of depression

**DOI:** 10.1007/s12035-016-9807-4

**Published:** 2016-03-02

**Authors:** Katarzyna Głombik, Aneta Stachowicz, Rafał Olszanecki, Joanna Ślusarczyk, Ewa Trojan, Władysław Lasoń, Marta Kubera, Bogusława Budziszewska, Michael Spedding, Agnieszka Basta-Kaim

**Affiliations:** 1 Department of Experimental Neuroendocrinology, Institute of Pharmacology, Polish Academy of Sciences, 12 Smętna St, 31-343 Kraków, Poland; 2 Chair of Pharmacology, Jagiellonian University Medical College, 16 Grzegórzecka Street, 31-531 Kraków, Poland; 3 Physiopathogie des Maladies Psychiatriques, INSERM UMR_S 894, Centre de Psychiatrie et Neurosciences, 2ter rue d’Alesia, 75014 Paris, France

**Keywords:** Tianeptine, Prenatal stress, Mitochondria, Hippocampus, Frontal cortex

## Abstract

A growing body of evidence has focused on the impact of mitochondrial disturbances in the development of depression, but little data exist regarding the effects of chronic administration of antidepressant drugs on the brain’s mitochondrial protein profile. The aim of this study was to investigate the impact of chronic treatment with an atypical antidepressant drug—tianeptine—on the mitochondria-enriched subproteome profile in the hippocampus and the frontal cortex of 3-month-old male rats following a prenatal stress procedure. Rats that were exposed to a prenatal stress procedure displayed depressive- and anxiety-like disturbances based on the elevated plus-maze and Porsolt tests. Moreover, two-dimensional electrophoresis coupled with mass spectrometry showed structure-dependent mitoproteome changes in brains of prenatally stressed rats after chronic tianeptine administration. A component of 2-oxoglutarate and succinate flavoprotein subunit dehydrogenases, isocitrate subunit alpha, was upregulated in the hippocampus. In the frontal cortex, there was a striking increase in the expression of glutamate dehydrogenase and cytochrome bc1 complex subunit 2. These findings suggest that mitochondria are underappreciated targets for therapeutic interventions, and mitochondrial function may be crucial for the effective treatment of stress-related diseases.

## Introduction

There exist well-established theories of depression that are based on monoamine transmission, immune system dysfunction, the weakened action of neurotropic factors, and hyperactivity of the hypothalamus-pituitary-adrenal (HPA) axis. Recently, another interesting theory called “the mitochondrial dysfunction hypothesis” has been highlighted [1, 2]. Impaired function of mitochondria leads to bioenergetic malfunctions, diminished adenosine triphosphate (ATP) production, impaired calcium homeostasis, and the enhanced production of free radicals and oxidative stress, leading to the initiation of apoptotic processes [3]. Furthermore, mitochondria in the central nervous system (CNS) may control processes of neuroplasticity, including neural differentiation, neurite outgrowth, dendritic remodeling, and neurotransmitter release [4]. Some data also suggested the interaction of mitochondrial function with epigenetic processes that are important in the pathogenesis of neuropsychiatric disorders, including depression symptoms [5]. All of these observations indicate that mitochondrial malfunctions may be a crucial factor in metabolic disturbances leading to depression [2].

In agreement with this theory, the following phenomena have been observed in depression: changes in mitochondrial morphology, decreases in mitochondrial respiration, increases in the polymorphisms in mitochondrial DNA (mtDNA) and the level of mtDNA mutations, as well as the downregulation of nuclear mRNAs and the proteins engaged in mitochondrial respiration. Moreover, electron microscopy data have demonstrated alterations in the number of mitochondria and their distribution in brain areas that are important in the pathogenesis of these diseases [6]. Decreased levels of high-energy phosphates and a diminished pH in the brains of depressive patients have generally been observed [7].

Therefore, mitochondria may be an interesting target for therapeutic intervention. An improvement in mitochondrial function could represent a crucial component in the effective treatment of stress-related diseases [8].

Tianeptine, an atypical antidepressant, seems to be most interesting and promising among the antidepressant drugs; however, the molecular mechanism of tianeptine action is still controversial. The neurochemical properties of tianeptine differ from those of other tricyclic and nontricyclic antidepressants. While classic tricyclic antidepressants and selective serotonin reuptake inhibitors (SSRI) block serotonin reuptake, tianeptine was shown to selectively enhance serotonin uptake into rat brain synaptosomes [9]. Tianeptine may also have properties related to its effect on the glutamatergic system and the reversal of stress-associated impaired neuroplasticity [10]. Moreover, the anticonvulsant and analgesic effects of tianeptine have been postulated [9]. So far, only a few in vitro studies have investigated the influence of tianeptine on isolated mitochondria [11]. Tianeptine inhibits complex I activity in vitro, but other mitochondrial complexes, apoptotic processes, and membrane potential reductions were not affected by this drug [3]. However, Della [12] observed in adult, maternally deprived rats that chronic tianeptine administration leads to a decrease in the creatinine concentration in the amygdala and the hippocampus and modulated the levels of mitochondrial complex I and complex II/III of the respiratory chain.

To the best of our knowledge, there are no data concerning the effect of chronic tianeptine treatment on the expression of the brain’s mitochondrial protein profile in an animal model of depression. The mitochondrial pathology in depression may result from, among other reasons, early adverse life experiences affecting brain development. Therefore, in the present study, we used one of the well-characterized animal models of depression, the prenatal stress procedure. In this model, behavioral disturbances and abnormalities in the functioning of the immune and neuroendocrine systems have been observed [13–18]. Furthermore, our previous study showed that stress during pregnancy may lead to disturbances in both mitochondrial biogenesis in the brain as well as in the mitoproteome in adult offspring [19].

In the present study, we applied two-dimensional electrophoresis coupled with mass spectrometry to investigate the influence of chronic administration of the atypical antidepressant drug tianeptine on the changes in the mitochondrial proteomic expression profile in the brain areas affected by prenatal stress and with major importance in the pathogenesis of depression, namely, the hippocampus and the frontal cortex of adult 3-month-old male rats.

## Materials and methods

### Animals

Sprague–Dawley rats (200–250 g upon arrival) were obtained from Charles-River Laboratories (Germany) and were maintained under standard conditions. Vaginal smears from the female rats were taken daily to determine the phase of the estrous cycle. On the day of proestrus, females were placed with males for 12 h and the presence of sperm in vaginal smears was confirmed the next morning. Pregnant females were randomly assigned to the control and stress groups (*n* = 8 per group).

All experiments were performed in accordance with the National Institutes of Health Guide for the Care and Use of Laboratory Animals and were approved by the Local Ethics Committee, Kraków, Poland (permit no. 699,18.01.2010).

### Stress procedure

The prenatal stress procedure was performed as described previously [13, 15, 17]. Briefly, the pregnant females were subjected to three stress sessions daily beginning on the 14th day of pregnancy and continuing until delivery. At 9:00 am, 12:00 pm, and 5:00 pm, the rats were placed in plastic cylinders (7/12 cm) and exposed to a bright light (150 W) for 45 min. Control pregnant females were left undisturbed in their home cages. Only offspring from litters containing eight to ten pups with a similar number of males and females were included in the study. Male offspring were selected for the experiment from 21-day-old litters. These rats were housed in groups of four animals per cage (one to two animals from each litter). At 3 months of age, the offspring of control and stressed mothers underwent the first behavioral verification.

### Elevated plus-maze test

The elevated plus-maze test was performed according to a method described previously by Pellow [20] in control and prenatally stressed rats (*n* = 18 each group). The maze was elevated to a height of 50 cm above the floor and illuminated only with a dim light from beneath. The animals were placed in the experimental room for 1 h before the test. Each animal was placed in the central area of the maze facing the closed arm and observed for a total of 5 min. The results are presented as the average number of entries into the open and the closed arms of the maze and the average time spent in each.

### Forced swim test (FST, Porsolt test)

For further verification of the animal model of depression, the forced swim test was conducted according to the method described previously [16, 21]. Briefly, the animals (*n* = 18 each group) were individually subjected to two trials, during which they were forced to swim in a cylinder filled with water (25 °C) to a height of 35 cm. There was a 24-h interval between the first and the second trial. The first trial lasted 15 min, and the second trial lasted 5 min. The total durations of immobility, mobility (swimming), and climbing were measured during the second trial [21, 22].

**Fig. 1:**
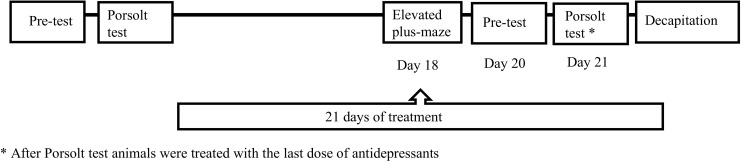
Schematic diagram representing the schedule of the experiment

For pharmacological verification of the animal model of depression, animals underwent the elevated plus-maze and forced swim procedure again on the last days of chronic antidepressant drug treatment (according to the schedule illustrated in Fig. 1).

### Antidepressant drug administration

After the behavioral verification, the control and prenatally stressed offspring were divided into six experimental groups (CONTROL + VEH, CONTROL + IMI, CONTROL + TIA, STRESS + VEH, STRESS + IMI, STRESS + TIA; six animals per group) and were treated for 21 days with antidepressant drugs or vehicle. Imipramine (used as a reference drug to compare tianeptine efficiency in behavioral tests; Sigma-Aldrich, St. Louis, MO, USA), tianeptine (Servier, France) or vehicle (0.9 % saline, Polpharma, Poland) was injected intraperitoneally, once per day between 9:00 am and 10:00 am. Imipramine and tianeptine were injected at a dose of 10 mg/ml/kg in a volume of 1 ml/kg. Twenty-four hours after the last injection, the animals were sacrificed by rapid decapitation. The hippocampi and the frontal cortices were dissected.

### Subcellular fractionation

A mitochondria-enriched fraction was isolated at 4 °C from freshly harvested brain structures. Brain structures were homogenized in a solution of 250 mM sucrose and 1 mM EGTA, pH 7.8 with the addition of 1 mM PMSF and a protease inhibitor mixture (Sigma, USA). Nuclei and unbroken cells were pulled down by centrifugation at 1000 *g* for 10 min. Then, the mitochondrial fraction was obtained by centrifugation of the supernatant at 12,000 *g* for 10 min. The mitochondria-enriched pellet was then purified using 3 cycles of resuspension, homogenization, and centrifugation (at 12,000 *g* for 15, 20, and 15 min). The cytosolic fraction was obtained by further centrifugation of the supernatant (90 min at 125,000 *g*, 4 °C).

### 2-DE and gel image analysis

Mitochondria-enriched pellets were resuspended in 0.5 ml of lysis buffer containing 7 M urea, 2 M thiourea, 4 % CHAPS, 1 % DTT, 0.5 % Bio-Lyte 3–10 (Bio-Rad, USA) and a protease inhibitor mix. Samples were vortexed and incubated at 25 °C for 30 min to ensure maximal protein solubilization; samples were then centrifuged at 12,000 *g* for 15 min. The supernatant was harvested, and the protein concentration was determined [23]. The supernatant was divided into aliquots containing an appropriate amount of protein for single IPG strips (300 μg for analytical gels). Samples were purified by precipitation using the two-dimensional electrophoresis (2-DE) Clean-up kit (GE Healthcare, USA) and resuspended in 450 μl of rehydration buffer (7 M urea, 2 M thiourea, 4 % CHAPS, 1.2 % DeStreak Reagent (GE Healthcare, USA), and 0.5 % Bio-Lyte 3–10). Then, samples were loaded onto 24-cm, nonlinear 3–10 immobilized pH gradient strips (Bio-Rad, USA) using an in-gel rehydration method and were rehydrated overnight in a reswelling tray. The strips were focused with a multistep voltage gradient of 150 to 6000 V (max 50 mA/IPG strip, 20 °C) for a total of 100 kVh. Once IEF was completed, the strips were equilibrated in buffer (6 M urea, 30 % glycerol, 2 % SDS, and 0.01 % bromophenol blue) with addition of 1 % *w*/*v* DTT (20 min) and 4.8 % *w*/*v* iodoacetamide (20 min) to ensure sufficient reduction and alkylation of proteins. The second dimension was obtained using a 9–16 % gradient SDS-PAGE gel without a stacking gel using the Protean Plus Dodeca Cell system (Bio-Rad, USA). After electrophoresis, the gels were fixed overnight in an ethanol/acetic acid/water solution (4:1:5 *v*/*v*/*v*). Finally, protein profiles were visualized by silver staining using the Plus One silver staining kit (GE Healthcare, USA) with modifications to provide compatibility with subsequent mass spectrometry analysis [24]. Silver-stained gels were imaged using a GS 800 densitometer (Bio-Rad, USA). PDQuest™ 8.0.1 (Bio-Rad, USA) was used for gel image analysis, quantification, and statistical validation. In total, *n* = 3 gel images representing each group from the total number of *n* = 2 replicates of two individual samples (*n* = 6 gel images per group) were analyzed. All results were carefully verified manually, including the verification of housekeeping proteins chosen for normalization (local regression model) to ensure accurate quantification. Significant differences were further analyzed with an LC MS/MS system to identify proteins of interest.

### LC MS/MS

Gel pieces containing protein spots of interest were destained, reduced, alkylated, and digested with modified trypsin (Promega) according to the protocol described by Shevchenko [25]. Peptide maps were lyophilized and stored at −80 °C for further LC/MS analysis. Each sample was resuspended in 0.1 % FA and injected onto an Acclaim PepMap100 RP C18 75 μm (i.d.) × 15 cm column (Thermo Scientific) via a trap column (Acclaim PepMap100 RP C18 75 μm (i.d.) × 2 cm column, Thermo Scientific). Peptides were separated over 55 min in a 7–55 % B phase linear gradient (A phase—2 % ACN and 0.1 % formic acid; B phase—80 % ACN and 0.1 % formic acid) with a flow rate of 300 ml/min using a Switchos/UltiMate 3000 HPLC system (LC Packings/Thermo Scientific) and applied on-line to a Velos Pro (Thermo Scientific) ion-trap mass spectrometer. The main working liquid-junction nanoESI ion source parameters were as follows: capillary voltage, 1.7 kV, and capillary temperature, 250 °C. Spectra were collected in full-scan mode (400–1500 Da); five MS/MS scans were then performed of the five most intensive ions from the full scan using dynamic exclusion criteria. Collected MS/MS data were analyzed by the X! Tandem search algorithm (The GPM Organization) and statistically validated with Peptide and Protein Prophet (Trans-Proteomic Pipeline, Institute for Systems Biology).

### Immunoblotting

The purity of the fractions was assessed by immunoblotting of cytochrome c oxidase (COX-IV) and cyclophilin A. The isolated mitochondria-enriched fraction was lysed in PBS containing 1 % Triton X-100, 0.1 % SDS, 1 mM PMSF, 100 μM leupeptin, and 50 μM pepstatin A. Samples containing equal amounts of total protein were mixed with gel loading buffer (50 mM Tris, 10 % SDS, 10 % glycerol, 10 % 2-mercaptoethanol, and 2 mg/ml bromophenol blue) in a 4:1 ratio (*v*/*v*) and incubated at 95 °C for 5 min. Samples were separated on SDS-polyacrylamide gels (Mini Protean II, Bio-Rad, USA) using the Laemmli buffer system, and proteins were semidry-transferred to nitrocellulose membranes (Amersham Biosciences, USA). Membranes were blocked overnight at 4 °C with 5 % nonfat dried milk in TTBS and incubated for 3 h at room temperature with specific primary antibodies: 1:5000 ANTI-COX4, 1:1:000 ANTI-Cyclophilin A (Abcam, USA). Membranes were then incubated for 1 h with HRP-conjugated secondary antibodies (Amersham Biosciences, USA). Bands were developed with the use of ECL-system reagents (Amersham Biosciences, USA). Protein pattern images were taken using a LAS-500 scanner (GE Healthcare, USA).

### Statistical analysis

The statistical analyses were performed with Statistica 10.0 software (Statsoft, Tulsa, USA). The outcomes of behavioral studies are presented as the mean ± SEM. The behavioral data were analyzed using one-way or two-way analysis of variance (ANOVA) with prenatal stress and treatment as the factors, and *p* values lower than 0.05 were regarded as statistically significant. The significance of differences between the means was evaluated by Duncan’s test. ANOVA assumptions (normality of variable distribution and homogeneity of variances) were assessed by the Shapiro-Wilk and Levene tests, respectively. The Student’s *t* test was used to reveal statistically significant differences in the expression of mitochondrial proteins.

## Results

### Impact of chronic tianeptine administration on the anxiety-like behavior of prenatally stressed rats

**Fig. 2:**
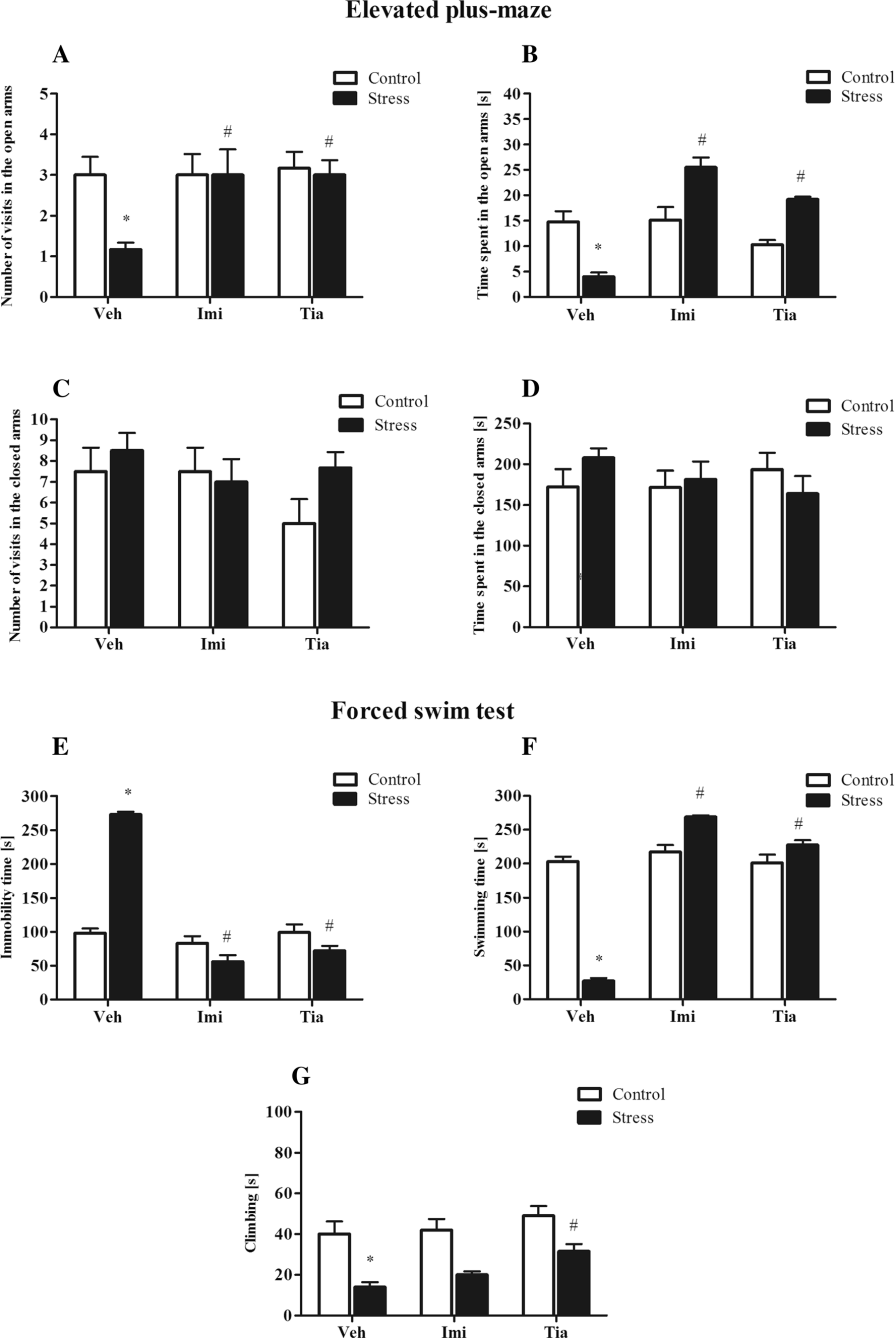
The effects of prenatal stress and chronic imipramine or tianeptine administration on the number of visits (**a**) and the time spent (**b**) in the open arms of the elevated plus-maze and the number of visits (**c**) and the time spent (**d**) in the closed arms of the elevated plus-maze. The effects of prenatal stress and chronic imipramine or tianeptine administration on the immobility (**e**), the swimming (**f**), and the climbing (**g**) times (in seconds) in the forced swim test. The data are presented as the means ± SEMs, with *n* = 6 for each group, ANOVA (two-way), followed by Duncan’s test. *p ≤ 0.05 compared with the control Veh group; #*p* ≤ 0.05 compared with the prenatally stressed Veh group

The elevated plus-maze test was used to assess anxiety-like behavior in rats. As expected, as shown in Table 1, the prenatal stress procedure caused a significant reduction in the number of entries into the open arms (*F*1,34 = 32.46; *p* < 0.05) of the maze and a significant decrease in the time spent in them (*F*1,34 = 88.57; *p* < 0.05). However, we did not observe differences in the number of entries to the closed arms and the time spent in them. Next, to determine whether chronic tianeptine or imipramine (as a reference drug to compare with tianeptine) administration affected the anxiety-like behavior evoked by the prenatal stress, we assessed the elevated plus-maze test again. As in the first set of experiments, in prenatally stressed rats, we observed a reduction in the number of entries into the open arms of the maze (*F*1,30 = 4.67) and a significant decrease in the time spent in them (*F*1,30 = 4.17). Moreover, we found a significant effect of drugs (*F*2,30 = 4.32; *p* < 0.05) and the stress × drug interaction (*F*2,30 = 3.60; *p* < 0.05) for the number of visits into the open arms (Fig. 2a) as well as a significant effect of drugs (*F*2,30 = 21.48; *p* < 0.05; Fig. 2a) and the stress × drug interaction (*F*2,30 = 25.14; *p* < 0.05; Fig. 2a, b) for the time spent in the open arms. Post hoc comparisons showed that both imipramine (*p* < 0.05) and tianeptine (*p* < 0.05) normalized the number of entries into the open arms of the maze and the time spent in them (*p* < 0.05).

**Table 1:** The effect of prenatal stress on the number of visits and the time spent in the open and the closed arms in the elevated plus-maze and on the times for immobility, swimming, and climbing in the forced swim test

	Elevated plus-maze	
	Control	Stress
Number of visits in the open arms	3.3 ± 0.3	1.5 ± 0.5*
Time spent in the open arm [s]	15.1 ± 1.0	5.2 ± 0.4*
Number of visits in the closed arms	7.5 ± 1.1	8.5 ± 0.8
Time spent in the closed arm [s]	180 ± 20.1	212 ± 10.0
Forced swim test		
Immobility [s]	99.4 ± 5.1	264.8 ± 4.7*
Swimming [s]	199.9 ± 5.1	35.2 ± 4.7*
Climbing [s]	40.2 ± 4.1	15.2 ± 1.9*

The results are presented as the mean ± SEM, *p < 0.05, *n* = 18 for each group, one-way ANOVA

### Impact of chronic tianeptine administration on the depressive-like behavior of prenatally stressed rats

To evaluate the depression-like behavior in rats, we performed the forced swim test (Porsolt test). We confirmed [16] that the prenatal stress procedure caused significantly prolonged immobility (*F*1,34 = 569.01; *p* < 0.05) and shortened swimming (*F*1,34 = 569.83; *p* < 0.05) and climbing (*F*1,34 = 29.95; *p* < 0.05) times (Table 1). In the next set of experiments, we determined the effects of chronic tianeptine and imipramine (as a reference) administration on the behavioral changes evoked by the prenatal stress by performing the Porsolt test again. We observed an increase in the immobility (*F*1,30 = 31.16; *p* < 0.05) and a decrease in swimming (*F*1,30 = 24.77; *p* < 0.05) and climbing (*F*1,30 = 25.76; *p* < 0.05) times, which confirmed that the changes induced by the prenatal stress procedure are long lasting (Fig. 2e, f, g). Furthermore, we observed a significant effect of the drugs (*F*2,30 = 99.43; *p* < 0.05; Fig. 2e) and the stress × drug interaction (*F*2,30 = 86.05; *p* < 0.05; Fig. 2e) on the immobility time. Post hoc comparisons revealed that imipramine (*p* < 0.05) and tianeptine (*p* < 0.05) shortened the immobility time in prenatally stressed offspring. Additionally, for swimming time, we observed a significant effect of drugs (*F*2,30 = 141.65; *p* < 0.05; Fig. 2f) and the stress × drug interaction (*F*2,30 = 121.18; *p* < 0.05; Fig. 2f). Post hoc comparisons revealed that imipramine (*p* < 0.05) and tianeptine (*p* < 0.05) extended the swimming time in the offspring of stressed rats. Importantly, only tianeptine (*p* < 0.05) prolonged the climbing time in prenatally stressed rats (Fig. 2g).

### Impact of chronic tianeptine administration on the mitochondria-enriched subproteome in the hippocampus and the frontal cortex of prenatally stressed rats

**Fig. 3:**
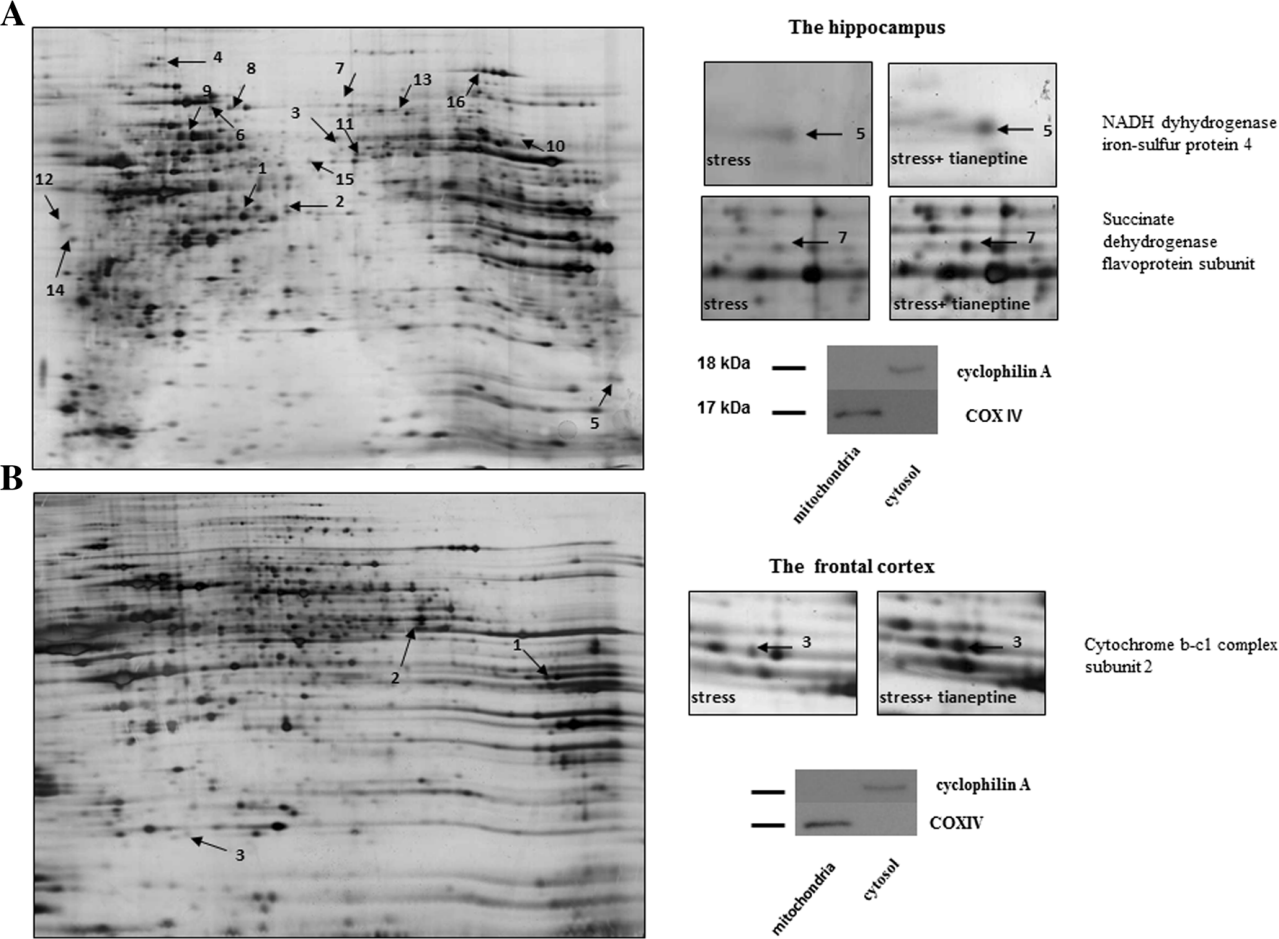
Representative 2-D map of the mitochondrial proteins expressed in the hippocampus (**a**) and the frontal cortex (**b**) of prenatally stressed rats treated with tianeptine compared with Those of prenatally stressed rats, with magnifications of spot pairs corresponding to NADH dehydrogenase iron-sulfur protein 4, succinate dehydrogenase flavoprotein subunit, and cytochrome b-c1 complex subunit 2. The purity of the mitochondrial fraction was assessed by Western blotting, which showed the absence of cytosolic cyclophilin A in the mitochondrial fraction

**Fig. 4:**
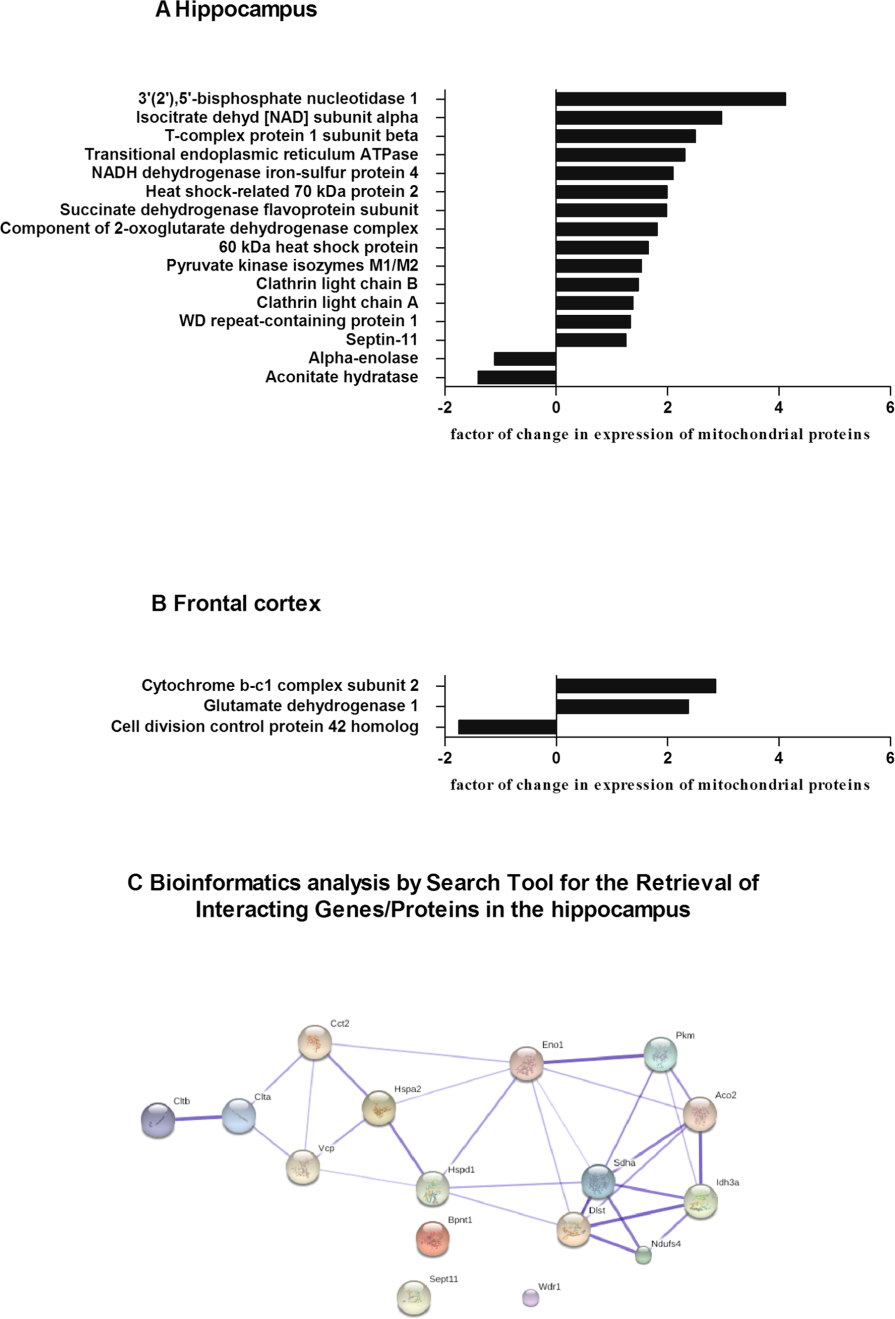
Relative changes in the expression of mitochondrial proteins in the prenatally stressed group treated with tianeptine compared with those of the prenatally stressed group in the hippocampus (**a**) and in the frontal cortex (**b**). Bioinformatics analysis by Search Tool for the Retrieval of Interacting Genes/Proteins in the hippocampus (**c**). The list of the identified proteins in the hippocampus was subjected to STRING (version 10) analysis to reveal functional interactions between the dysregulated proteins. *Each node* represents a protein, and *each edge* represents an association. Stronger associations are represented by *thicker lines*

We investigated the influence of tianeptine on mitochondrial protein expression in the selected brain areas using 2-DE coupled with tandem mass spectrometry. The representative 2-DE gel images of the mitochondrial proteins from the prenatally stressed animals treated with tianeptine, as well as selected pairs of spots showing the differences between the prenatally stressed offspring and prenatally stressed animals treated with tianeptine, are presented for the hippocampus and the frontal cortex (Fig. 3). The LC MS/MS analysis from the numbered spots and the associated differences are listed in Table 2. Quantitation of the significant differences in the expression of the mitochondrial proteins is shown in Fig. 4a, b. The accuracy of the isolation protocol and the purity of the mitochondrial fractions were assessed by immunoblotting for cyclophilin A and COX-IV (Fig. 3).

**Table 2:** Differentially expressed proteins in the hippocampus (A) and the frontal cortex (B) of prenatally stressed rats treated with tianeptine compared with prenatally stressed rats

No.	Protein	UniProtKB accession number	Molecular mass (Da)	pI^a^	Unique peptides	Total peptides	Protein coverage (%)	Fold change
(A) Hippocampus								
1	3′(2′),5′-bisphosphate nucleotidase 1 (Bpnt1)	Q9Z1N4	33,153	5.58	5	21	17.9 %	4,12
2	Isocitrate dehyd [NAD] subunit alpha (Idh3a)	Q99NA5	39,588	6.46	3	6	7.9 %	2,97
3	T-complex protein 1 subunit beta (Cct2)	Q5XIM9	57,422	6.01	9	18	16.8 %	2,5
4	Transitional endoplasmic reticulum ATPase (Vcp)	P46462	89,293	5.14	11	30	17.0 %	2,31
5	NADH dehydrogenase iron-sulfur protein 4 (Ndufs4)	Q5XIF3	19,728	10.14	2	8	11 %	2,1
6	Heat shock-related 70 kDa protein 2 (Hspa2)	P14659	69,599	5.51	2	11	3.8 %	1,99
7	Succinate dehydrogenase flavoprotein subunit (Sdha)	Q920L2	71,570	6.75	5	34	9.2 %	1,98
8	Component of 2-oxoglutarate dehydrogenase complex (Dlst)	Q01205	48,894	8.89	4	17	7.0 %	1,81
9	60 kDa heat shock protein (Hspd1)	P63039	60,917	5.91	15	39	32.3 %	1,65
10	Pyruvate kinase isozymes M1/M2 (Pkm)	P11980	57,781	6.63	8	18	22 %	1,53
11	Clathrin light chain B (Cltb)	P08082	25,102	4.56	5	14	17.9 %	1,47
12	Clathrin light chain A (Clta)	P08081	26,964	4.41	3	37	10.5 %	1,38
13	WD repeat-containing protein 1 (Wdr1)	Q5RKI0	66,140	6.15	6	20	10.4 %	1,33
14	Septin-11 (Sept11)	B3GNI6	49,663	6.24	5	9	10.2 %	1,25
15	Alpha-enolase (Eno1)	P04764	47,098	6.16	4	13	14.1 %	−1,1
16	Aconitate hydratase (Aco2)	Q9ER34	85,380	7.87	9	33	14.90 %	−1,4
(B) Frontal cortex								
1	Cytochrome b-c1 complex subunit 2	P32551	48,366	9.16	17	41	40.70 %	2,86
2	Glutamate dehydrogenase 1	P10860	61,377	8.05	26	69	50.40 %	2,37
3	Cell division control protein 42 homolog	Q8CFN2	21,245	6.16	3	5	17.80 %	−1,75

Briefly, in the hippocampus, 16 differentially expressed spots were detected in the samples from prenatally stressed rats treated with tianeptine. As shown in Table 2, the expression of 14 proteins was increased; these increased proteins included metabolic enzymes (e.g., isocitrate dehydrogenase [NAD] subunit alpha, +2.97-fold change; NADH dehydrogenase iron-sulfur protein 4, +2.1-fold change; succinate dehydrogenase flavoprotein subunit, +1.98-fold change; and a component of the 2-oxoglutarate dehydrogenase complex, +1.81-fold change) and heat shock proteins (HSP70, +1.99-fold change and HSP60, +1.65-fold change). In the frontal cortex of the prenatally stressed offspring that underwent chronic tianeptine administration, we identified only three differentially expressed spots using PDQuest™, which were analyzed by mass spectrometry. Among them, two spots were upregulated (cytochrome b-c1 complex subunit 2, +2.86-fold change and glutamate dehydrogenase 1, +2.37-fold change) and one was downregulated (cell division control protein 42 homolog, −1.75-fold change).

Protein clusters in the hippocampus and the frontal cortex were identified according to GO Annotation by the Search Tool for the Retrieval of Interacting Genes (STRING, version 10) database (http://string-db.org) using “molecular function,” “biological processes,” or “cellular component” as a criterion. The STRING database integrates interaction data from several bioinformatics sources and provides information about physical and functional properties as well as known and predicted interactions of genes and their products. The analysis did not show any correlation between proteins in the frontal cortex. However, in the hippocampus, an analysis using molecular function as a criterion revealed a strong correlation between eight proteins. Furthermore, in the hippocampus, correlations of all proteins were observed using cellular component as a criterion, clearly confirming the purity of the samples that were subjected to proteomic analysis. In Fig. 4c, we show a screenshot from the bioinformatics analysis and the associations between proteins in the hippocampus.

## Discussion

Recent studies [2] have noted that the brain functions at the limit of its energy supply, which can limit neuronal activity. Furthermore, trophic stimuli such as brain-derived neurotrophic factor (BDNF) increase brain mitochondrial efficiency; however, this effect is blocked by inflammatory cytokines [26], which can contribute to depression. Mitochondrial disease leads to debilitating fatigue [27], mitochondrial DNA changes, and oxidative damage have been reported in patient cohorts with depression [28]; therefore, mitochondrial function may be compromised in some forms of depression.

Our study demonstrated, for the first time, that chronic administration of the atypical antidepressant drug tianeptine not only normalized the behavioral disturbances in adult prenatally stressed animals but also affected the protein expression profile in brain mitochondria. These effects involved the critical rate-limiting enzymes in the Krebs cycle, predominantly in the hippocampus and to a lesser extent in the frontal cortex of adult male offspring.

The prenatal stress procedure is a well-established animal model of depression, whose predictive, face and construct validity have already been documented [29, 30]. In the present study, the behavioral tests were used for verification of tianeptine efficiency, while imipramine, a tricyclic antidepressant drug, was applied only as a reference drug for comparison with the behavioral efficacy of tianeptine. We confirmed that adult male rats born to dams exposed to stress during the third trimester of pregnancy displayed a prolonged immobility time in the forced swimming test and shortened swimming and climbing times [15]. The present study showed that chronic tianeptine administration normalized all of the parameters evaluated in the Porsolt test. Furthermore, tianeptine administration attenuated the anxiety-like behavior evoked by prenatal stress as assessed by an increase in the number of entries and in the time spent in the open arms of the maze.

Chronic treatment with tianeptine modulated mitoproteomic changes in the hippocampus and the frontal cortex after the prenatal stress procedure [19]. A growing body of evidence suggests that mitochondrial disturbances play a key role in the pathogenesis of depression [19, 31], but the impact of chronic tianeptine administration on the mitochondria-enriched subproteome in both the hippocampus and the frontal cortex in an animal model of depression had not been investigated so far. With regard to the hippocampus, the upregulated expression of isocitrate dehydrogenase (IDH) after chronic tianeptine administration appears to be the most striking finding of our study. So far, there are no data showing the impact of antidepressants on the mitochondrial expression of this rate-limiting Krebs cycle enzyme. Importantly, our data demonstrated that chronic tianeptine administration in prenatally stressed offspring increased the expression of a component of the 2-oxoglutarate dehydrogenase complex (OGDHC). The OGDHC comprises multiple copies of three catabolic components, the action of which is required for an important regulatory step in the mitochondrial Krebs cycle (the oxidative decarboxylation of 2-oxoglutarate, which generates energy in the form of NADH and the macroergic compound succinyl-CoA) [32]. Some data demonstrated that malfunction of the OGDHC associated with mitochondrial dysfunction might be a crucial point in the pathogenesis of depression [33]. Consistent with this finding, the stimulation of the brain OGDHC complex by a synthetic analog of 2-oxoglutarate upregulated the activity of this enzymatic complex and affected behavioral changes, which were expressed as an increase in exploratory activity and diminished anxiety behavior in rats [32]. Based on this observation, it may be postulated that the ability of tianeptine to upregulate the OGDHC in the hippocampus may have a beneficial impact on the brain metabolism and on the behavioral disturbances observed in prenatally stressed animals.

Remarkably, the dehydrogenases that were affected by tianeptine are the key rate-limiting enzymes in the Krebs cycle. Demetrius [34] has noted the key role of switching between oxidative phosphorylation and glycolysis in neurodegeneration, where Krebs cycle activity is critical. Isocitrate dehydrogenase expression is diminished in a mouse model of Alzheimer’s disease, and the administration of an antisense nucleotide targeting β-amyloid reduced oxidative stress-induced damage, possibly by an increased expression of isocitrate dehydrogenase [35].

Chronic tianeptine administration enhanced the expression of succinate dehydrogenase (SDH), an enzyme important not only for the Krebs cycle but also in the mitochondrial respiratory chain as an electron-transferring protein [36]. In addition, SDH activity is one of the most reliable markers of the efficiency of mitochondrial ATP production, and altered activity of this enzyme leads to impaired brain energy metabolism [37]. Most data indicated that chronic stress in rats decreased the activity of SDH in the brain [38]. Thus, the increase in SDH expression after tianeptine treatment will have a beneficial impact on brain metabolism. Moreover, our data are consistent with previously published reports that chronic treatment with antidepressants, such as nortriptyline, paroxetine, or venlafaxine, enhances SDH activity in the brain [8]. In the hippocampus, chronic tianeptine treatment of our model of depression upregulated not only Krebs cycle enzymes but also proteins in the respiratory chain including SDH as well as NADH dehydrogenase iron-sulfur protein 4 [39].

A further finding of the present study is the observation that tianeptine administration increased the expression of heat shock proteins HSP60 and HSP70 in the hippocampus of prenatally stressed offspring. These conserved proteins fulfill a protective function, thus eliminating harmful effects, including stress. Zlatković [40] showed that HSP70 upregulation protects the hippocampus from chronic social isolation stress. HSP70 limits the accumulation and aggregation of misfolded proteins by directing them to the proteosomal degradation pathway [41, 42]. HSP60 also has a role in protection from proteinopathy [43].

Interestingly, in contrast to the hippocampus, the frontal cortex of adult 3-month-old prenatally stressed offspring was susceptible to a limited extent to tianeptine action because its administration changed the expression profile of only three mitochondrial proteins; among these changes was the upregulation of glutamate dehydrogenase 1 (GDH1). The link between depressive-like behavior and reduced GDH1 expression has been observed in the brains of bipolar patients [44]. GDH1 facilitates the oxidative transamination of glutamate into alpha-ketoglutarate [45]; thus, the upregulation of GDH1 by chronic tianeptine treatment may target the Krebs cycle in the frontal cortex of prenatally stressed offspring.

Our study also demonstrated that the expression of cytochrome b-c1 complex subunit 2 (also known as complex III or cytochrome ubiquinone oxidoreductase) was significantly enhanced in the mitoproteome in the frontal cortex of prenatally stressed rats after tianeptine administration. Complex III catalyzes the transfer of electrons from reduced coenzyme Q to cytochrome c associated with proton pumping to the intermembrane space [46]. Up until now, the data relating to the action of antidepressants on the respiratory chain have been inconclusive. Hroudova and Fisar [47] showed that chronic administration of various antidepressants and mood stabilizers suppressed not only complex III but also complexes I, II, and IV of the respiratory chain in brain homogenates of untreated pigs. Investigations in adult male rats revealed that chronic immobilization stress inhibited the activity of complex III of the mitochondrial respiratory chain without affecting complex IV activity, ATP production, or oxygen consumption [48]. Importantly, the impact of tianeptine administration on the activity of the respiratory chain seems to be dependent on the brain structure and the duration of treatment [12, 49]. The limitation of our study is that it is a mitoproteomic analysis after chronic tianeptine treatment in a model of depression; nevertheless, the present results point to a new molecular mechanism of tianeptine that involves the modulation of metabolic changes.

In summary, our findings clearly demonstrate that chronic tianeptine administration effectively attenuated the behavioral disturbances provoked by early adverse life experiences. Moreover, 2-DE coupled with mass spectrometry showed for the first time that chronic tianeptine administration ameliorated disruptions in the hippocampal mitoproteome in an animal model of depression. The key proteins were a component of the 2-oxoglutarate dehydrogenase complex, isocitrate dehydrogenase [NAD] subunit alpha, energy production enzymes (succinate dehydrogenase flavoprotein subunit and NADH dehydrogenase iron-sulfur protein 4), and stress-related proteins (HSP60 and HSP70). Furthermore, in the frontal cortex, we noted the upregulation of proteins related to the glutamate pathway and the respiratory chain in prenatally stressed rats chronically treated with tianeptine. Overall, the directions of tianeptine-induced changes in the mitoproteome are opposed to those observed in models of depression or stress-related disorders.

To conclude, mitochondria may be a critical target for therapeutic interventions in psychiatry, and the improvement of mitochondrial function could be crucial for the effective treatment of stress-related diseases.
